# Supplementary material for: Reduction of variability for the assessment of side effects of toxicants on honeybees and understanding drivers for colony development
Source: PLoS One. 2020 Feb 14;15(2):e0229295. doi: 10.1371/journal.pone.0229295 (PMC7021311; doi:10.1371/journal.pone.0229295)
Supplement: S1 File — (PDF) [file pone.0229295.s001.pdf]

Supplementary material

Wang, M., Braasch, T. and Dietrich, C. Reduction of variability for the assessment of side effects of toxicants on honeybees and understanding drivers for colony development.
Submitted Aug. 2019.

1. Method to measure flight activity and forager mortality using video recordings

Programmable video cameras were placed above the entrance of hives (Figure 1). A white landing board is used to facilitate the distinction of bees from the background. Videos were recorded from dawn to dusk and subsequently analysed in the software VideoCounter 1.1. In the software individual bees are recognized in videos and their movement path is tracked. A transect is used to define the direction into which bees fly (in the hive or outside of the hive). To exclude bees flying above the hive entrance, the recognition of flying bees was restricted based on the size of bees.

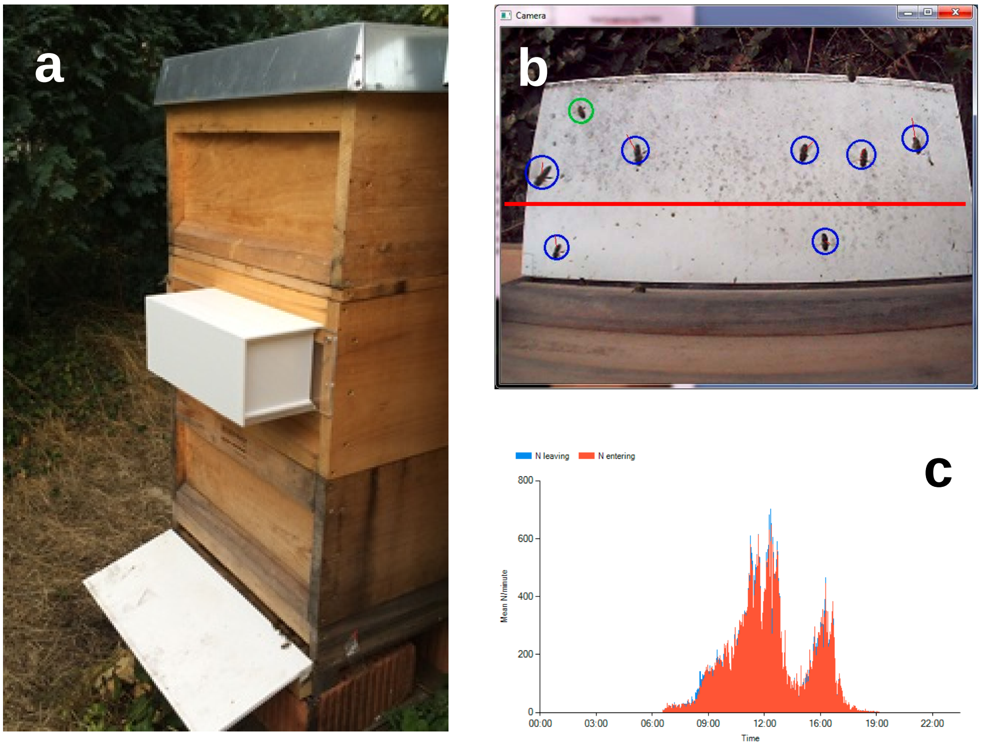

Figure 1: a) Video camera mounted above the hive entrance. b) Example of a video in which bees are recognized. c) Example of flight activity during one day.

The software was previously validated regarding the accuracy of counting (which was 108.7% for bees entering the hive and 82.7% for bees leaving the hive; resolution 320 x 240 px) and correction factors were applied to obtain corrected counts. Counts reflect foraging activity. From these counts forager mortality can be calculated by subtracting the daily number of entering bees from the number of leaving bees.

2. Detailed results of colony strength

Colony strength was estimated by weight (Table 1), photography of adult bees (Table 2) and by visual estimation (Liebefelder), as recommended by OECD 75 (Table 3).

Table 1: Mean and standard deviation (SD) of the number of adult obtained from colony weight measurements.

Date	10.04.	13.04.	24.04.	28.04	02.05.	07.05.	13.05	19.05	25.05	1.06.
**Means [N]**										
Control	13500	16850	25625	32100	30725	28625	29600	29600	31400	23000
Reference	16275	21325	31025	37575	29525	28300	30375	31025	25775	25925
**Standard deviations (SD) [N]**										
Control	3524	3339	1970	2637	763	1144	1359	5026	5940	2064
Reference	2065	2259	2056	3353	4549	3008	5273	7020	3291	4849
**Means [% of initial number]**										
Control	100.0	124.8	189.8	237.8	227.6	212.0	219.3	219.3	232.6	170.4
Reference	100.0	131.0	190.6	230.9	181.4	173.9	186.6	190.6	158.4	159.3
**Standard deviations [% of initial number]**										
Control	26.1	19.8	7.7	8.2	2.5	4.0	4.6	17.0	18.9	9.0
Reference	12.7	10.6	6.6	8.9	15.4	10.6	17.4	22.6	12.8	18.7

Table 2: Mean and standard deviation (SD) of automated counts of adult bees of hives and hive walls in photos.

Date	07.04.	14.4.	21.04.	28.04	03.05.	08.05.	14.05.	20.05.	26.05.	02.06.
**Means [N]**										
Control	9508	10183	13857	14567	16083	15226	16298	18050	16208	15705
Reference	12783	14372	17734	17785	15347	15382	18192	20216	16767	16064
**Standard deviations (SD) [N]**										
Control	1378	1955	1698	1334	1508	2408	2062	2273	2240	2033
Reference	1317	1771	973	1755	1825	1509	2789	2601	2088	2504
**Means [% of initial number]**										
Control	100.0	107.1	145.7	153.2	169.1	160.1	171.4	189.8	170.5	165.2
Reference	100.0	112.4	138.7	139.1	120.1	120.3	142.3	158.2	131.2	125.7
**Standard deviations [% of initial number]**										
SD in Control	14.5	19.2	12.3	9.2	9.4	15.8	12.7	12.6	13.8	12.9
SD in Reference	10.3	12.3	5.5	9.9	11.9	9.8	15.3	12.9	12.5	15.6

Table 3: Mean and standard deviation (SD) of adult counts obtained from visual estimations.

Date	07.04.	14.04.	21.04.	28.04.	03.05.	08.05.	14.05.	20.05.	26.05.	02.06.
**Means [N]**										
Control	10094	9625	13375	14313	15500	16281	18375	19000	15906	14313
Reference	14219	14469	16813	16875	15906	16094	20188	19906	15281	15531
**Standard deviations (SD) [N]**										
Control	1569	2013	1468	1023	1717	2864	1785	1591	2831	1379
Reference	1416	1473	1175	2556	2357	2959	3217	3068	3412	2548
**Means [% of initial number]**										
Control	100.0	95.4	132.5	141.8	153.6	161.3	182.0	188.2	157.6	141.8
Reference	100.0	101.8	118.2	118.7	111.9	113.2	142.0	140.0	107.5	109.2
**Standard deviations [% of initial number]**										
Control	15.5	20.9	11.0	7.1	11.1	17.6	9.7	8.4	17.8	9.6
Reference	10.0	10.2	7.0	15.1	14.8	18.4	15.9	15.4	22.3	16.4

3. Impact of the application of the reference substance on forager mortality obtained from dead bee traps, sheets and video analysis

The number of dead counted either in dead bees traps is shown in Figure 2. The underlying data and counts in traps and sheets are provided in Table 4 and 5. Forager mortality obtained by video analysis of bees leaving and entering the hive is presented in Table 6.

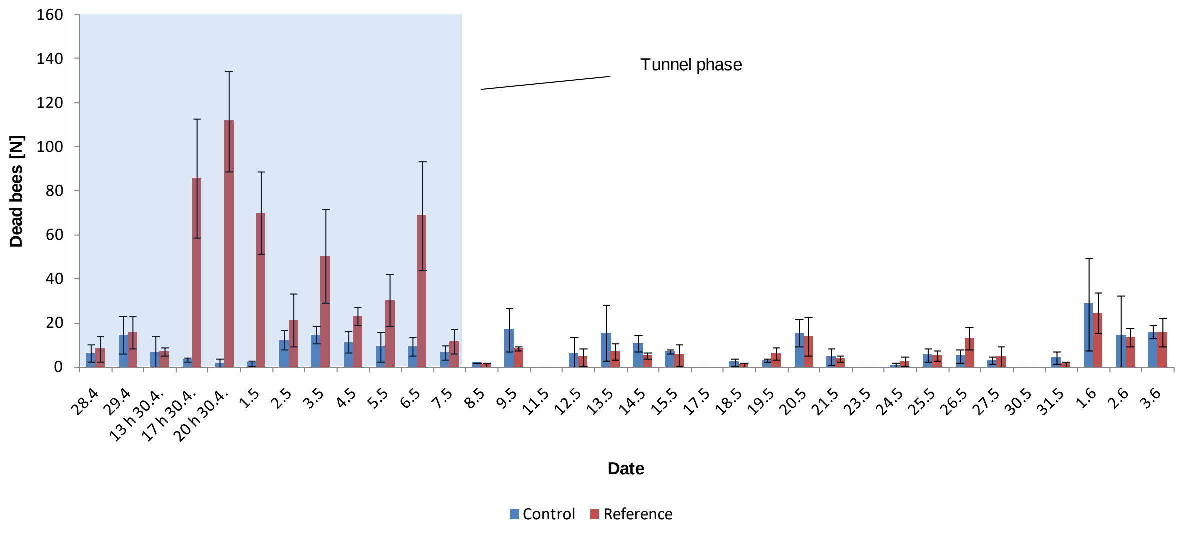

Figure 2: Number of dead bees (workers, drones and pupae) collected in dead bee traps in the control and reference. Error bars indicate one standard deviation.

Table 4: Dead bee counts [N] from dead bee traps in the control and reference.

Date/time	Assess-ment	Control				Reference				Dead bees [% of control]
		Workers	Pupae	Drones	Sum	Workers	Pupae	Drones	Sum	
28.04.2017	1	23	0	2	25	32	0	1	33	132
29.04.2017	1	56	1	1	58	60	1	2	63	109
30.04.2017 13 h	1	17	0	10	27	28	0	0	28	104
30.04.2017 17 h	2	8	0	5	13	343	0	0	343	2638
30.04.2017 20 h	3	2	0	4	6	447	0	0	447	7450
30.04.2017	1-3	27	0	19	46	818	0	0	818	1778
01.05.2017	1	6	1	0	7	280	0	0	280	4000
02.05.2017	1	44	4	1	49	83	0	2	85	173
03.05.2017	1	49	6	3	58	198	2	2	202	348
04.05.2017	1	38	5	2	45	90	1	2	93	207
05.05.2017	1	27	6	4	37	110	7	4	121	327
06.05.2017	1	32	5	1	38	259	13	3	275	724
07.05.2017	1	25	0	1	26	46	0	1	47	181
08.05.2017	1	7	0	1	8	3	1	0	4	50
09.05.2017	1	49	16	3	68	21	8	4	33	49
12.05.2017	1	23	0	1	24	9	0	9	18	75
13.05.2017	1	58	2	2	62	25	2	1	28	45
14.05.2017	1	39	2	2	43	20	0	0	20	47
15.05.2017	1	27	0	1	28	18	1	3	22	79
18.05.2017	1	9	0	0	9	3	1	0	4	44
19.05.2017	1	12	0	0	12	25	0	0	25	208
20.05.2017	1	60	0	2	62	56	0	0	56	90
21.05.2017	1	18	0	0	18	15	0	0	15	83

Date/time	Assess-ment	Control				Reference				Dead bees [% of control]
		Workers	Pupae	Drones	Sum	Workers	Pupae	Drones	Sum	
24.05.2017	1	3	0	0	3	10	0	0	10	333
25.05.2017	1	22	0	0	22	21	0	0	21	95
26.05.2017	1	20	0	0	20	51	0	1	52	260
27.05.2017	1	12	0	0	12	18	0	1	19	158
31.05.2017	1	16	0	1	17	5	0	0	5	29
01.06.2017	1	111	0	3	114	98	0	0	98	86
02.06.2017	1	57	0	0	57	47	0	7	54	95
03.06.2017	1	63	0	1	64	56	1	6	63	98

Table 5: Dead bee counts [N] on sheets in the control and reference.

Date/time	Assess-ment	Control				Reference				Dead bees [% of control]
		Workers	Pupae	Drones	Sum	Workers	Pupae	Drones	Sum	
28.04.2017	1	33	0	0	33	72	0	0	72	218
29.04.2017	1	119	0	0	119	194	0	0	194	163
30.04.2017 13 h	1	46	0	0	46	59	0	0	59	128
30.04.2017 17 h	2	9	0	0	9	27	0	0	27	300
30.04.2017 20 h	3	0	0	0	0	46	0	0	46	-
30.04.2017	1-3	55	0	0	55	132	0	0	132	240
01.05.2017	1	15	0	0	15	50	0	0	50	333
02.05.2017	1	95	0	0	95	182	0	0	182	192
03.05.2017	1	120	0	1	121	262	0	2	264	218
04.05.2017	1	108	0	0	108	187	0	2	189	175
05.05.2017	1	211	0	0	211	164	0	0	164	78
06.05.2017	1	226	0	12	238	316	0	4	320	134
07.05.2017	1	40	0	0	40	40	0	0	40	100

Table 6: Daily forager mortality of control and reference hives during different phases of the study, counted by automated video analysis.

Phase	Pre-/post-application	Mean daily forager mortality [%]		Standard deviation of daily forager mortality [%]	
		Control	Reference	Control	Reference
**Tunnel phase**	Pre-application	13.0	10.5	2.7	2.0
	Post-application	14.5	17.7	7.6	3.7
**Post-tunnel phase**		2.5	4.3	3.7	2.4

4. Impact of no. of cells used for brood evaluation

Introduction

The aim of this assessment was to understand to what extent brood termination rate (BTR) depends on the number of cells that are evaluated. I.e. the uncertainty related to sample size was analysed.

Methods

Monte Carlo simulations were conducted using the brood development data of a hive from the group treated with the reference substance (hive 17-5). In this hive brood termination rate (BTR) was 83.1% for cells which contained eggs at the time of brood fixing (two days before application, DAA-2). The total number of cells with eggs at brood fixing day 0 (BFD0) was 3418. From these either 100 or 300 brood cells were randomly chosen and brood termination rates were calculated for the resulting subsets of cells. This process was repeated 1000 times to obtain an overview of how much brood termination rate can vary depending which cells are chosen.

Results

When evaluating 100 cells starting with eggs at BFD0, BTR ranged from 65.0% to 97.0%. When choosing 300 cells starting with eggs at BFD0, BTR ranged from 73.3% to 91.3%. In comparison, the true BTR of this colony was 83.1%.

5. Parameters considered for the evaluation of factors influence brood termination

To understand which factors affect brood termination generalized linear models (GLMs) were used. Since most termination occurred after the egg stage BTR_{egg} was analyzed. The following parameters were taken into account:

Table 7: List of parameters considered for generalized linear models (GLMs) describing BTR_{egg}.

Considered parameters for GLMs
Adults from weight (AFW)
Pollen cells (PC)
Nectar cells (NC)
Young larvae cells (YL)
Capped brood cells (CB)
Open brood cells (OB)
Pollen cells per young larvae (PCYL)
Pollen cells per old larvae (PCOL)

Model parameter estimates are provided in the following for each model (model numbers and parameter abbreviations according to Table 2 in manuscript):

Table 8: Standardized model parameter estimates of generalized linear models (GLM) describing BTR_{egg}. Negative parameter estimates indicate a negative influence of BTR_{egg}, positive parameter estimates indicate a positive influence of BTR_{egg}. Due to the standardization single parameters can easily be compared to each other, while the estimate of combined parameters and quadratic terms should be considered as being on another scale.

Model	Parameter	Estimate	Std. Error	z value	Pr(>|z|)
1	CB	1.9685	0.5374	3.663	**0.0002**
	OB	-2.1848	0.5233	-4.175	**< 0.0001**
	Constant	-1.1145	0.4050	-2.752	**0.0059**
2	CB	2.1056	0.5005	4.207	**< 0.0001**
	OB	1.4991	1.6360	0.916	0.3595
	OB^2	-4.0101	1.6664	-2.406	**0.0161**
	Constant	-1.8476	0.4688	-3.942	**< 0.0001**
3	CB	4.4299	0.8968	4.940	**< 0.0001**
	OB	-1.5617	0.5644	-2.767	**0.0057**
	PC	3.9888	1.3929	2.864	**0.0042**
	CB*PC	-7.6067	2.4002	-3.169	**0.0015**
	Constant	-2.7963	0.6392	-4.375	**< 0.0001**

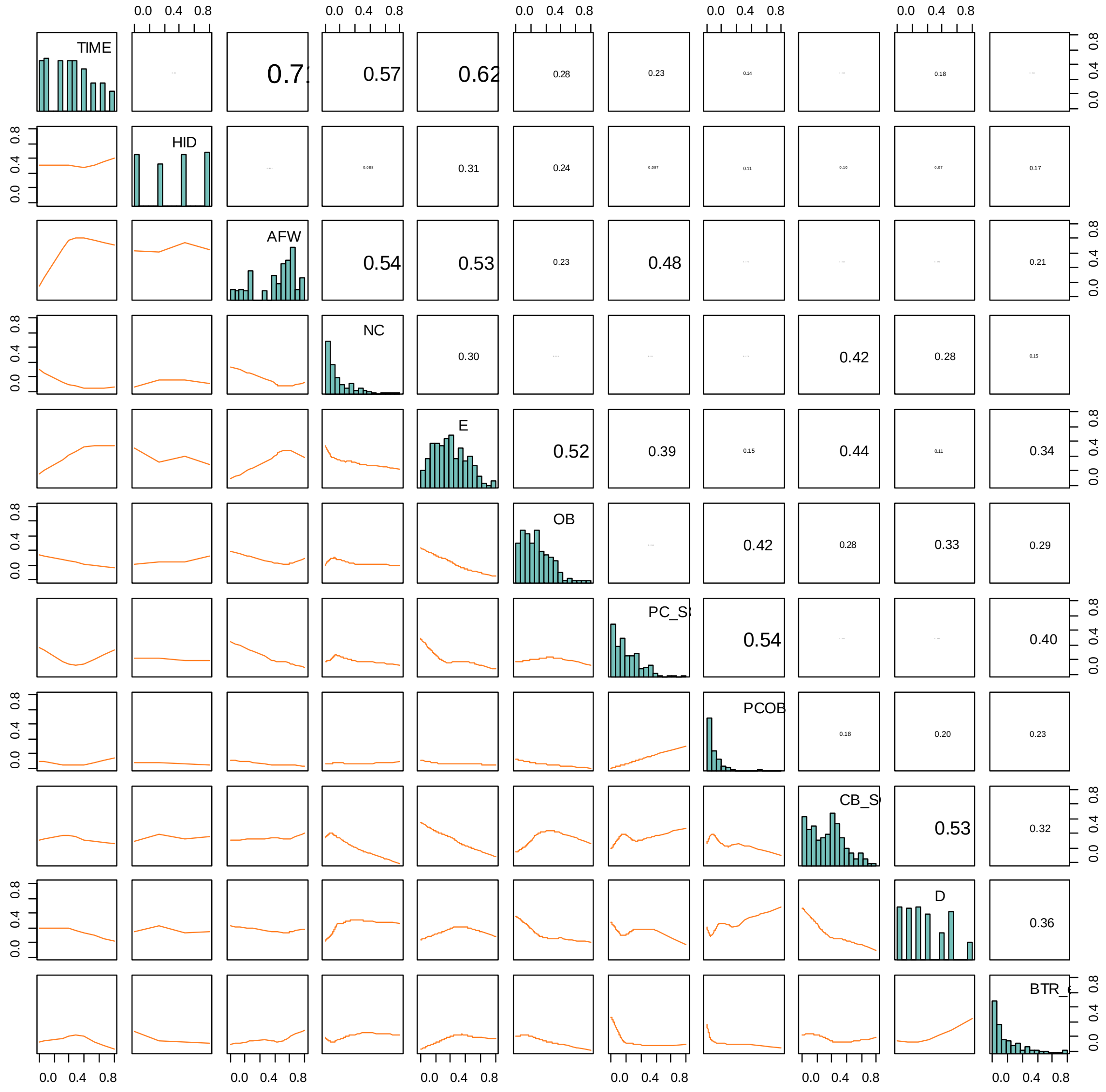

Figure 3: **Pair-wise relations between measurements in the hives. The main diagonal shows histograms of the single standardized variables. The upper triangular matrix shows correlation coefficients. The bigger the number is displayed, the greater is the correlation coefficient. Note the weakly pronounced correlation between the BTR_{egg} and the time (TIME) and the hive index (HID) and the main drivers of BTR_{egg} namely the number of pollen cells on the same or opposite frame (PC_SOF), the distance from the hive center (D), the number of empty cells (E) and the number of capped brood on the same or opposite frame (CD_SOF). The lower triangular matrix shows a simple smoothed line referring to the relationship of each two variables. Remaining variables are the number of adults from weight (AFW), the number of nectar cells (NC), the number of open brood cells (OB) and the number of pollen cells per open brood cell (PCOB).**

6. Calculation of power curves for colony size

To illustrate which sample sizes would be required to reach specific MDD values for colony size power curves (Fig. 7 in publication) were calculated using Monte Carlo randomization. Notably, the calculation of these sample sizes is somewhat theoretical, since in practice, sample sizes of more than six to eight hives are not used in OECD 75 trials. However, results illustrate the effort needed to detect effects of a given magnitude.

Calculations were conducted either assuming a random choice of colonies (named 'Conventional trial' in the following[1]) for the tunnel phase or by considering the data distributions of the actually selected hives (named 'LUV trial' in the following). The analysis was done for the endpoints adults as measured by weight, photography and visual estimations and assuming normal distribution. To estimate distributions resulting from a random choice of colonies, the coefficients of variation (CV_{weight} = 16.12%, CV_{photo} = 20.96%, CV_{visual} = 22.24%) measured for all hives on the last day before the selection of hives were considered (as only the selected hives were further evaluated after the selection). To estimate distributions of selected hives (LUV trial) coefficients of variation measured on the first day in the tunnel were used (control: CV_{weight} = 8.2%, CV_{photo} = 9.2%, CV_{visual} = 7.1%; reference: CV_{weight} = 8.9%, CV_{photo} = 9.9%, CV_{visual} = 15.1%). As a mean of the normal distributions the average number of adults measured on the first measurement day in the tunnels (28.4.) was used to reflect a situation when a pesticide would be applied. The resulting means and standard deviations are shown in Table 9. These were used for conduct Monte Carlo simulations using 1000 iterations to calculate MDD according to Brock et al. (2014).

Table 9: Means and standard deviations of colony strength (no. adult bees) used for Monte Carlo simulations to calculate power curves.

	No. adults (weight)		No. adults (photography)		No. adults (visual)	
	Mean	SD	Mean	SD	Mean	SD
Conventional trial (random selection)[1]	34838	5616	16176	3391	15594	3462
LUV trial, control	32100	2637	14567	1334	14313	1023
LUV trial, reference	37575	3353	17786	1755	16875	2556

[1] Used for both control and reference, since at the time when all hives were monitored hives had not yet been assigned to groups. Mean values were based on the CV on the last measurement day before placing hives in the tunnels and the mean of selected hives on the first measurement day in the tunnel.

Results are shown in Figure 7 of the publication.

[1] Note that all colonies had already been chosen to be similar at the time when they were obtained from the bee keeper. Hence the choice of hives followed the same procedure as in a conventional trial.

7. Comparison of study design in comparison to a conventional OECD 75 trial

Table 10: Schematic comparison of study design used in the present study in comparison to a conventional OECD 75 trial.

	Present study	Conventional OECD 75 trial
Pre-tunnel phase	Monitoring of colony weight, mortality, hive content (brood and food) over four weeks<br><br>Methods used (for comparison):<br>• Colony strength by weight, photography and visual estimation<br>• Hive content (brood and food) by photography of all combs of all hives<br>• Mortality: Dead bee traps	-
Selection of colonies	Selection of colonies based on development during pre-tunnel phase (colony strength, mortality, capped brood), sister queens	Use of similar or equalized colonies by visual estimation, sister queens
Tunnel phase	Statistical comparison of test groups (no test substance used, only reference substance, hence no test done in this generic study; clear effects visible)<br><br>Methods used (for comparison):<br>• Colony strength by weight, photography and visual estimation<br>• Brood success: Evaluation of all brood cells<br>• Food content: Evaluation of all cells of all hives<br>• Flight activity: Visual count in three 1 m² for seconds or minutes, videography over the entire flight period<br>• Mortality: Dead bee traps, linen sheets calculation of forager mortality from videography	Statistical comparison of test groups<br><br>Methods:<br>• Colony strength: Visual estimation<br>• Brood success: 100-200 brood cells<br>• Food content: Visual estimation<br>• Flight activity: Visual count in three 1 m² for seconds or minutes<br>Mortality: Dead bee traps, linen sheets
Post-tunnel phase	Same as in tunnel phase (except for use of linen sheets)	Same as in tunnel phase (except for use of linen sheets)
