# Supplementary material for: Reduction of variability for the assessment of side effects of toxicants on honeybees and understanding drivers for colony development
Source: PLoS One. 2020 Feb 14;15(2):e0229295. doi: 10.1371/journal.pone.0229295 (PMC7021311; doi:10.1371/journal.pone.0229295)
Supplement: Attachment — Submitted filename: Response to Reviewers.docx [file pone.0229295.s002.docx]

**Response to reviewers**

**Journal Requirements:**

1. When submitting your revision, we need you to address these additional requirements.

*Reply: We have taken these formatting requirements into account.*

2. We note that one or more of the authors are employed by a commercial company: WSC Scientific GmbH.

a) Please provide an amended Funding Statement declaring this commercial affiliation, as well as a statement regarding the Role of Funders in your study. If the funding organization did not play a role in the study design, data collection and analysis, decision to publish, or preparation of the manuscript and only provided financial support in the form of authors' salaries and/or research materials, please review your statements relating to the author contributions, and ensure you have specifically and accurately indicated the role(s) that these authors had in your study. You can update author roles in the Author Contributions section of the online submission form.

Please also include the following statement within your amended Funding Statement.

“The funder provided support in the form of salaries for authors [insert relevant initials], but did not have any additional role in the study design, data collection and analysis, decision to publish, or preparation of the manuscript. The specific roles of these authors are articulated in the ‘author contributions’ section.”
If your commercial affiliation did play a role in your study, please state and explain this role within your updated Funding Statement.

*Reply: We amended the financial disclosure statement as follows:*

*This research was funded entirely by the commercial company WSC Scientific GmbH. The funder provided support in the form of salaries for authors [MW, TB, CD] and materials, but did not have any additional role in the study design, data collection and analysis, decision to publish, or preparation of the manuscript. The specific roles of these authors are articulated in the ‘author contributions’ section.*

b)  Please also provide an updated Competing Interests Statement declaring this commercial affiliation along with any other relevant declarations relating to employment, consultancy, patents, products in development, or marketed products, etc.

Within your Competing Interests Statement, please confirm that this commercial affiliation does not alter your adherence to all PLOS ONE policies on sharing data and materials by including the following statement: "This does not alter our adherence to  PLOS ONE policies on sharing data and materials.” (as detailed online in our guide for authors http://journals.plos.org/plosone/s/competing-interests) . If this adherence statement is not accurate and  there are restrictions on sharing of data and/or materials, please state these. Please note that we cannot proceed with consideration of your article until this information has been declared.

Please include both an updated Funding Statement and Competing Interests Statement in your cover letter. We will change the online submission form on your behalf.

*Reply: We added the following Competing Interest Statement (we don’t expect a commercial benefit from this study, as industry is not interested in refined methodology (which may improve the detection of adverse effects of pesticides), however, academia or governmental authorities may be interested in an improvement of the method, which would, however, not result in any commercial benefit of the funding company):*

*[MW, TB, CD] are employed at WSC Scientific GmbH, which, amongst others, develops scientific software including software for the evaluation of honeybee trials. The purpose of this study was, however, not to promote such software, which contributes only marginally to the turnover of the company, but to develop better study design for the risk assessment. This does not alter our adherence to PLOS ONE policies on sharing data and materials.*

3. We note that you have included the phrase “data not shown” in your manuscript. Unfortunately, this does not meet our data sharing requirements. PLOS does not permit references to inaccessible data. We require that authors provide all relevant data within the paper, Supporting Information files, or in an acceptable, public repository. Please add a citation to support this phrase or upload the data that corresponds with these findings to a stable repository (such as Figshare or Dryad) and provide and URLs, DOIs, or accession numbers that may be used to access these data. Or, if the data are not a core part of the research being presented in your study, we ask that you remove the phrase that refers to these data.

*Reply: We have added these data in the Supplemental Material now and replaced “data not shown” with ”S1 Fig. 2, Tables 4-6”.*

**Comments to the Author**

5. Review Comments to the Author

Reviewer #1: This study reports the results of a semi-field trial where caged colonies of honeybees (n = 4) are exposed to either exposure to an insecticide or control conditions. The authors use the data from the experiment to evaluate the power of the experimental design to detect treatment effects. The authors claim that the novel protocols used in their experiment enable fairly small treatment effects to be detected, which might be able to meet the levels of resolution stipulated in recent European regulations.

The experiments and data analysis appear to be technically sound and the paper is fairly well-written.

The main implication – that adjusted protocols will enable semi-field trials to detect pesticidal effects at the levels required by EU regulations – will be very important and interesting to a wide audience, including regulators, industry and environmentalists.

There are, however, some fairly major shortcomings that should be addressed if the wok is to reach its full impact, which I describe as follows.

1. Setting the baseline

It would be very useful to set the context – what is the MDD of previous semi-field trials? In the discussion, some evaluation of the level of improvement should be given.

*Reply: We added a section in which we discuss a paper by Rolke et al. (2016), in which MDD was calculated (the use of MDD is a relatively new concept in pesticide risk assessment). Using twice as many colonies MDD ranged between 15.2–21.4% compared to about 10% in our study with only four colonies.*

2. Power analysis

One of the conventional components of a power analysis is an effort-power curve, which is the relationship between the MDD and sample size across a continuous range. Here it would be useful to see the curves from n = 2 to n = 20. In discussion, it would be useful to compare the power curves of the old protocols with the authors’ new protocols. Curves should be presented for all response variables.

*Reply: Such a power curve would indeed be helpful. However, since honeybee trials according to OECD 75 are done with three to six colonies per test group at most, such data is not available (such studies are very cost intensive even with these sample sizes). We are not aware of any (published or unpublished) study with more than six colonies per test group.*

3. Interpretation of causality

It is not correct to attribute the reduction in MDD over time to the selection of the hives because it might have decreased in any case. Justifying this assertion would require comparing the MDD when hives were picked from a pool of all hives versus the MDD when hives were picked from a reduced pool. Either do this or do not make the assertion.

Reply: We thank the reviewer for highlighting this. We added the information which MDD would have been reached without the selection procedure at the end of section “Pre-monitoring” in the results:

*“We also compared MDD of colony strength by weight on the day of selection of colonies under the assumption of randomly sampling four colonies for each test group by Monte Carlo analysis. When randomly choosing hives MDD was on average 21.6%. I.e. the selection procedure achieved a reduction of MDD by a factor of about two (10.8% by the selection procedure vs. 21.6% by randomly choosing hives).”*

4. Discussion

The current discussion is off-target. The main headline should be the improvement in MDD relative to past practice, which variables are most reliable, etc. The large opening section evaluating the various Delaplane methods does not warrant the space.

*Reply: We shortened the section where the work by Delaplane was discussed considerably.*

5. Data provision

There should be a table of means and SDs in the MS itself so that others can verify the power analyses.

*Reply: We added this information to the Supplementary Information S1 (Tables 1-3).*

Minor edits by line number
81-84: link to general theory by using some standard terms such as sampling, response variable.

*Reply: We don’t understand this comment.*

90: decreased, surely?

*Reply: Thank you, yes it needs to be “decreased”.*

100: clarify locations of phases 1-3

*Reply: Thank you for highlighting this, it is indeed clearer if we mention the locations more clearly for phases 1 and 3 (for the tunnel phase this was already done). We added the location in parenthesis for phases 1 and 3 now.*

109: dissected = eliminated

*Reply: We replaced ‘dissected’ with ‘eliminated’*

386: ‘very small’ is subjective – currently the reference point is ‘almost negligible’ at 7%

*Reply: In the previous sentence we stated that effects of 10% were detectable with N=4, hence we feel it is fine to name these ‘very small’ (as the absolute value was provided). However, to make clear that we do not reach 7% yet with N=4 we changed the sentence to ‘rather small’.*

Page 29: Figs 1 and 2 are indentical in my copy

*Reply: We corrected this now.*

Reviewer #2: The manuscript by Wang et al proposed and tested a new test design for risk assessment (RA) of pesticides in the field and semi-field, addressing major concerns.
The manuscript seems scientifically sound, mostly interesting for a relatively narrow community as the topic is very specific but with important broader outcome and global relevance as it proposes changes to the internationally used RA system. The MS could be more concise and clear, in terms of methodology and results/discussion. I think the MS is valuable and worth publishing in PlosONE after referee comments will be addressed. I highlight some major concerns that I believe, when addressed, could make the manuscript clearer, more impactful and robust.

*please address the CONS (negative aspects) related to your proposal, not only the pros. This is essential to estimate its feasibility and show a honest approach. i.e. increased costs in terms of expenses, time, organization, staff, etc.

Reply: We added a paragraph highlighting the CONS of the applied methodology in the discussion:

*“Using a larger number of hives and adding a monitoring phase to finally select a subset of colonies for the actual test does, however, increased the duration and workload for such a study by about a month. In particular the use of photography to count adult bees required a much larger number of field staff, since adult bee photography was done simultaneously for all hives to reduce bias by changing weather conditions. However, results have shown that colony size estimation by weight maybe a more cost effective alternative method with a similar level of measurement bias compared to photography.”*

*the proposed and current protocol should be reported in a table for clarity and ease of comparison. this tab can for ex. list the endpoints measured and how they are measured (time, etc) in your and the current system. (eg line 45-46, 33, 52,

Reply: Such a table is already included in the supplemental information (Section “4. Comparison of study design in comparison to a conventional OECD 75 trial”), there the differences are compared for all endpoints measured. If the editor prefers we could move this table from the supplementary information to the manuscript?

*improve clarity of the methods/proposal: use multiple subchapters for each step i.e. in line 107 and after.

Reply: We thank the reviewer for this excellent proposal. We have added a section before this line to first clarify which endpoints have been measured in which phase:

*“Throughout the whole study the following endpoints were measured (see below for details on the methods): colony strength, content of all cells of all hives (incl. brood, nectar, pollen), dead bees and larvae in traps. Foraging activity by visual assessment was only conducted in the tunnel phase and forager activity and mortality measurements by videography were only conducted in the tunnel and post-tunnel phase.”*

Furthermore we added a header “Step 1: Four week monitoring phase” and added a section for the following phase: “Step 2: A ten-day tunnel phase” and “Step 3: Post-tunnel phase”. We believe this does indeed help very much to clarify how the study was conducted.

*"approximately" is often used (L214, 162, and many more). A more clear and exact description of your protocol is needed as this is required by guidances and gives solidity to your proposal. Add frequency of assessment times for all steps (ie L 174-176)

Reply: We rephrased this section as follows (bold letters indicate changed text):

Line 162: ***“Measurements of*** *flight activity* ***were planned*** *in weekly intervals during and after the tunnel phase using video recordings of the entrances of all hives over the entire activity phase (from dawn to dusk; see supplementary material).* ***Actual dates varied by one to two days depending on weather conditions.”***

Line 214: *“Over a period of 32 days they were assessed approximately weekly* ***(depending on weather conditions)*** *to finally select eight very similar colonies […]”*

*SPG: how about the subelthal effects? Nonetheless you test forager activity etc, all other sublethal effects pesticides can cause are not addressed very much in your paper. This is another major concerns related to bee health, RA, SPGs. I'd clarify this and eventually state that your work goal does not include this specifically.

Reply: We believe it is sufficiently clear that we do not address sublethal effects when discussing the specific protection goal (SPG) defined by EFSA, because whenever ‘SPG’ is mentioned the context is provided:

Line 404 in original manuscript: *“With regard to the Specific Protection Goals (SPGs) for forager mortality […]”. Here it is clear that this only focuses on forager mortality.*

Line 409: *“Hence, this method more directly generates the data required to address the SPGs regarding forager mortality defined by EFSA (2013)”* Same as above.

If the editor prefers we would of course be happy to add an additional sentence mentioning that sublethal effects are not considered.

*clarify if you did field, semif, or both (ie. L90). certain basic details of your work should be easier to figure out. For ex did you test both scenarios with pre selection of colonies and not (L 212+)? If not you cannot compare the change in vairability.

Reply: Since the selection process would be the same for both field and semi-field trials, this sentence applies to both types of studies. We did, however, conduct only a semi-field study after the selection. But note that variability was reduced already before colonies were in the tunnels. In the sentence before it was already mentioned that we only did a semi-field study. However, to avoid any doubt we modified the sentence as follows:

*“Results* ***from our semi-field study*** *demonstrate that variability of both field and semi-field trials can be decreased significantly with the proposed new test design and methodology.”*

*colony exclusion procedure (L 219) needs to be described and showed explicity in terms of methods and results. an explicit method for excludsion decision needs to be used and described (ie. decision threshold for each endpoint ie. varroa, etc?). This is a crucial point that RA needs to clarify explicitly and objectively. please refer to previous guidelines if available on the topic.

Reply: We have added a more detailed description of the selection process in the methods section under the subheading “Selection of colonies at the end of the monitoring phase”. This section now reads as follows:

*“From these eleven colonies eight colonies were selected and randomly assigned to the control and the reference group (four colonies per group). The selection had the aim of achieving colonies of similar strength during the tunnel phase. This same rationale is used in many laboratory toxicity trials, where very ‘similar’ animals, e.g. of similar age and strain are selected in order to decrease variability (and increase test-power). This was achieved based on the development of colony size throughout the pre-tunnel monitoring phase (as measured by weight), the number of capped brood cells per hive and similar mortality (measured in dead bee traps). Specifically, the selection was done in two steps: 1. Exclusion of colonies due to other reasons than colony size (e.g. high varroa count, untypically high mortality in dead bee traps). 2. Comparison of colony size during the monitoring phase, i.e. the most similar eight colonies in terms of colony strength were selected and assigned randomly to the two test groups.”*

*the authors should address, briefly and concisely and explicitly in the discussion, how it was demonstrated, providing the key compartive quantitative values, that the LUV test improved the current standards (i.e. increase power); see lines 462-463. Please also explicitly report how you re results demonstrate that your assessment is more robust.

Reply: We modified the sentence:

*“Concluding, with the presented LUV test, it was possible to considerably increase test-power.”*

To:

*“Concluding, with the presented LUV test, it was possible to considerably increase test-power (as reflected by MDD, which was reduced from a values >20% to 10.8% after selection of colonies).”*

*major problems of field studies are not addressed: ie. the absence of real control colonies (i.e. pesticide-free) in the field (i.e. Campbell et al 2016 and Henry et al 2015 showed that control colonies were contaminated by the target pesticide too, and Tosi et al 2018 showed that the majority of the colonies in the environment are exposed to individual and multiple pesticides, even banned ones). I would at least mention this aspect and other key concerns related to field studies for RA.

Reply: While the issues mentioned by the reviewer are certainly an important point to consider in the risk assessment scheme in general, we feel that it is a bit out of the scope of this article. We could add a sentence such as:

*“As for all field or semi-field trials, there are still more issues to resolve for future testing, such as the use of truly unexposed control colonies (Henry et al., 2015; Campbell et al., 2016; Tosi et al., 2018). However, since colonies usually always have access to agricultural land or gardens when they are established before a field or semi-field test, it may be difficult to achieve this.”*

However, we feel it would be a bit lost in the discussion. If the editor prefers, we’d of course be happy to add at the end of the discussion.

LINE BY LINE COMMENTS:
abstract: spell out MDD

Reply: We did as suggested.

line 80. this was addressed before too. move above?

Reply: The methods above, described e.g. by Delaplane and Harbo (1987), differ to the ones we used. Delaplane and Harbo (1987) suggested to ‘create’ new colonies in order to achieve similar colonies, while we do not alter the colonies, but select the most similar ones. Therefore, we feel it is better to leave these parts of the text separately.

88 specify field or semifield?

Reply: We added “semi-field” to be more precise.

412-415 video counts: you reported it causes higher variability. Please address this in this section and evetual other CONS.

Reply: This is probably a misunderstanding. We wrote that “the forager activity assessments as proposed in OECD (2007) [1] do not cover the whole forager activity of a colony but only a fraction limited by time and space.“ I.e. we consider the assessment using videos to be much more precise than the very short, visual assessments of only a few 1m² squares proposed by OECD.

416-418. I imagine there would be others, I'd double check.

Reply: We did not find any papers on studies in which entire colonies (all cells) were evaluated. There were only papers on methods to count capped cells (e.g. Colin et al., 2018).

Colin T, Bruce J, Meikle WG, Barron AB 2018. The development of honey bee colonies assessed using a new semi-automated brood counting method: CombCount. PLOS ONE: https://dx.plos.org/10.1371/journal.pone.0205816

discussion: text is very long, i think it should be shorter and more concise.

Reply: We tried to shorten the discussion, specifically paragraph 1.

463-464: authors should be more careful when stating this, as you report results from 1 study, testing limited colonies and over a limited time frame (1 year). standard procedures for proposing new methods is ring testing them, ie. perfomed in multiple countries over multiple years. Thus, this statement seems not supported by your results.

Reply: We agree. We rephrased the paragraph and also added a sentence highlighting that so far this has been demonstrated only in one study (words in bold letters have been added):

*“Concluding, with the presented LUV test, it was possible to considerably increase test-power (as reflected by MDD, which was reduced from a values >20% to 10.8% after selection of colonies). This ~~does not only~~* ***may*** *make it now possible to empirically determine Specific Protection Goals (e.g. the seven percent effect size regarding colony strength) recently proposed by EFSA [3, 7] using expert judgement, but also to test the toxicity of chemicals with a much higher certainty. Furthermore, new insights ~~can~~****could*** *be gained regarding the impact of weather and other biotic or abiotic factors can be studied in greater detail.* ***However, as the results summarized above are based only on one study, further testing may be required to verify the findings of this study****.”*

figures: fig. 3: cannot see error bar in black bars.

Reply: We modified the figures with error bars so that also negative bars are now visible.

References
Campbell, J. W., Cabrera, A. R., Stanley-Stahr, C. & Ellis, J. D. An evaluation of the honey bee (Hymenoptera: Apidae) safety profile of a new systemic insecticide, flupyradifurone, under field conditions in Florida. J. Econ. Entomol. 96, 875–878 (2016).
Henry, M. et al. Reconciling laboratory and field assessments of neonicotinoid toxicity to honeybees. Proc. R. Soc. B Biol. Sci. 282, 20152110 (2015).Tosi, S., Costa, C., Vesco, U., Quaglia, G. & Guido, G. A 3-year survey of Italian honey bee-collected pollen reveals widespread contamination by agricultural pesticides. Sci. Total Environ. 615, 208–218 (2018).

6. PLOS authors have the option to publish the peer review history of their article ([what does this mean?](https://journals.plos.org/plosone/s/editorial-and-peer-review-process#loc-peer-review-history)). If published, this will include your full peer review and any attached files.


If you choose “no”, your identity will remain anonymous but your review may still be made public.


**Do you want your identity to be public for this peer review?** For information about this choice, including consent withdrawal, please see our [Privacy Policy](https://www.plos.org/privacy-policy).

Reviewer #1: No

Reviewer #2: No


[NOTE: If reviewer comments were submitted as an attachment file, they will be attached to this email and accessible via the submission site. Please log into your account, locate the manuscript record, and check for the action link "View Attachments". If this link does not appear, there are no attachment files to be viewed.]


While revising your submission, please upload your figure files to the Preflight Analysis and Conversion Engine (PACE) digital diagnostic tool, <https://pacev2.apexcovantage.com/>. PACE helps ensure that figures meet PLOS requirements. To use PACE, you must first register as a user. Registration is free. Then, login and navigate to the UPLOAD tab, where you will find detailed instructions on how to use the tool. If you encounter any issues or have any questions when using PACE, please email us at [figures@plos.org](mailto:figures@plos.org). Please note that Supporting Information files do not need this step.
