# Supplementary material for: Reduction of variability for the assessment of side effects of toxicants on honeybees and understanding drivers for colony development
Source: PLoS One. 2020 Feb 14;15(2):e0229295. doi: 10.1371/journal.pone.0229295 (PMC7021311; doi:10.1371/journal.pone.0229295)
Supplement: Attachment — Submitted filename: Response to Reviewers.docx [file pone.0229295.s003.docx]

**Response to reviewers**

**Reviewer #1: Your revisions are largely satisfactory and I think it will be a valuable contribution to this under-investigated and important topic. I have one request that was not addressed and I point out some minor edits.**

**1. Power curve - as previously requested**

**Since you are able to use Monte Carlo randomization, it should be straightforward to estimate power curves by sampling from parametric distributions with the same means and variances as the observed data. Or use the method that you used to estimate the effect of doubling the sample size (lines 410-413). Obviously, it would be nice to generate these empirically, but you can go a long way using your present observation.**

***Reply: Thank you for this suggestion, we thought you had meant to do a power analysis with actual data. Using Monte Carlo instead is a good idea. We added power curves as suggested as Figure 7 to the main text in the location proposed by the reviewer. Details to the methods used are shown in the supplementary materials (section 6).***

**The really valuable contribution here is to estimate the number of hives needed to detect 7% effects currently specified by the Specific Protection Goals and to indicate the likely number of hives needed to detect any given effect size.**

***Reply: We agree, this is now shown in Figure 7.***

**Minor edits by line number:**

**32: at driving?**

***Reply: Thank you, this is a typo it must be “and driving”***

**57: forager**

***Reply: We changed this to “foragers”***

**68: certainly not 'impossible' - 'logistically demanding', perhaps**

***Reply: As we refer to evaluations by EFSA of data that were generated in the past, we think that it is correct to state that it is impossible to detect small effects using these data. The reviewer probably means that in principle one could detect small effects with more effort. We would agree, but this is not meant here.***

**77: comma after colonies**

***Reply: Thank you, we added a comma.***

**143: 'toxicant application' - even 'reference substance' is too specialist jargon for this journal**

***Reply: We rephrased the sentence as follows: “Toxicant application (reference substance) was conducted two days after the start of the tunnel phase.”***

**209: based on**

***Reply: Thank you, we added “on”***

**258: define the pool that the selection was made from (the 11 colonies with or without replacement - with, surely?)**

***Reply: We added “with replacement” in backets.***

**266: t-tests of the effect of toxicant exposure on ..**

***Reply: We added “the effect of toxicant exposure on” as suggested.***

**275 - see 143**

***Reply: We have rephrased the sentence as follows (but we feel it is less clear now, the term ‘reference group’ is a term that all ecotoxicologists will be familiar with): “After the selection of the colonies, four hives were randomly assigned to each the control group and the group in which a toxicant was applied (reference group), respectively and they were placed into the tunnels.”***

**286 - as 143**

***Reply: We have replaced “control” with “control group”.***
